# Determining whether weight status mediates the association between number of cigarettes smoked per day and all-cause mortality among US adults who smoke cigarettes

**DOI:** 10.1371/journal.pone.0319560

**Published:** 2025-04-30

**Authors:** Luis Miguel Mestre, Roger S. Zoh, Cydne Perry, Julia Fukuyama, Maria A. Parker

**Affiliations:** 1 Department of Psychiatry, Yale School of Medicine, New Haven, Connecticut, United States of America; 2 Department of Epidemiology and Biostatistics, Indiana University School of Public Health- Bloomington, Bloomington, Indiana, United States of America; 3 Department of Applied Health Sciences, Indiana University School of Public Health- Bloomington, Bloomington, Indiana, United States of America; 4 Computing and Engineering Department of Statistics, Indiana University School of Informatics, Bloomington, Indiana, United States of America; Wuhan Fourth Hospital, CHINA

## Abstract

**Introduction:**

While there is evidence demonstrating the association between cigarette smoking and weight status, and mortality and weight status, it has not been examined whether weight status is a mediator between number of cigarettes smoked per day (CPD) and all-cause mortality, limiting our knowledge of this association and potential novel approaches to reduce all-cause mortality due to cigarette smoking. We aimed to evaluate whether weight status mediated the association between CPD and mortality.

**Methods:**

We harnessed the 2003-2018 NHANES and the Linkage Mortality Files, which included adults who smoked ≥ 100 lifetime cigarettes (unweighted n = 5,676). A generalized linear model estimated the association between cigarettes smoked per day (CPD) and weight status (e.g., Body Mass Index (BMI) or Waist Circumference (WC)). An Accelerated Failure Time model with a Weibull distribution estimated the association between CPD and all-cause mortality with weight status as a mediator, adjusting for age, SES, alcohol consumption, race/ethnicity, sex/gender, blood pressure, total cholesterol, and physical activity.

**Results:**

Between 2003-2018, the sample’s mean BMI was 27.97 kg/m^2^, sample’s mean WC was 97.58 cm and mean CPD was 13.21. The total effect in the mediation analysis of WC adjusted by BMI levels in the association between CPD and all-cause mortality was -0.44 (95% CI =  -2.00, -0.20; p =  0.016), the average direct effect was -0.35 (95% CI =  -1.86, -0.10; p =  0.036), and the average indirect effect was -0.10 (95% CI =  -0.23, -0.05; p < 0.001).

**Conclusion:**

WC, as a surrogate measure of weight status, when adjusted by BMI levels, was a partial mediator between CPD and all-cause mortality. Public health interventions aimed to reduce mortality due to cigarette smoking at the population level should consider weight management programs as a harm reduction strategy to reduce mortality.

## Introduction

Based on recent statistics, more than 28 million United States (US) adults currently smoke cigarettes [1], while more than 35% have obesity [2], and approximately 2% are classified as underweight [3]. Cigarette smoking and weight status are considered primary public health concerns [4] and major causes of preventable deaths in the US [5]. Although the association between cigarette smoking and weight status is well known [6–27], it is not entirely understood [21], which makes it difficult to prevent death due to combined cigarette smoking and weight status. Multiple studies have shown different directions and magnitudes of the association between cigarette smoking and weight status, especially when using a surrogate measures of weight status such as Body Mass Index (BMI) [6–14,16,18–21].

Individuals who smoke cigarettes tend to have a lower BMI than those who do not smoke cigarettes [6–12,15,19]. However, among those who smoke cigarettes, their waist circumference (WC), and thus central obesity, tend to be higher than those who do not smoke cigarettes [6,11,16–26]. Some studies have found that those who smoke less than 20 CPD (i.e., non-heavy smoking) have a lower BMI than those who do not smoke cigarettes; those who smoke 20 or more CPD (i.e., heavy smoking) have a higher BMI than those who smoke non-heavily or do not smoke cigarettes [6–8,20,21]. This is evidence of a U-shaped association between number of cigarettes smoked per day and BMI at the population level [7]. The reason why individuals who smoke 20 or more CPD tend to have a higher BMI than those with non-heavy smoking or who do not smoke remains unclear [7,11,21]. An accepted hypothesis is that those who heavily smoke also engage in other unhealthy behaviors [12], such as higher alcohol consumption and lower physical activity, behaviors associated with higher levels of BMI [25,28–30]. There is also evidence [10,16,17,22] of a positive dose-response relationship between the CPD and weight status when measured as WC; the higher the CPD, the higher the WC of the person who smokes cigarettes [17,22]. Thus, there is an association between cigarette smoking and weight status, especially central obesity, when approximated as WC [20,23,24,27].

It is well known that cigarette smoking [31–38] and weight status [39–53] are associated with all-cause mortality due to being two of the main risk factors of chronic diseases [35,36,38,45,46,48] and chronic inflammation [11,48]. Individuals who smoke cigarettes have at least a two-fold increasing risk of all-cause mortality [31,34–37] and lose at least ten years of life expectancy [38] than those who do not smoke cigarettes. Among those who smoke cigarettes, there is a dose-response relationship between the number of cigarettes smoked and the risk of all-cause mortality; the higher the number of cigarettes smoked, the higher the risk of all-cause mortality [32,33], even if the smoking behavior is nondaily, or < 1 CPD [33].

Prior studies have shown that individuals who are underweight (BMI <  18.5 kg/m^2^) or have obesity (BMI ≥  30 kg/m^2^) experience a higher risk of all-cause mortality than those with a normal weight or who are overweight [39,41–47,50]. In other words, BMI and all-cause mortality have a U-shaped relationship [39,43,44,46,50,54]. Such a U-shaped relationship is moderated by race/ethnicity and sex/gender, indicating that physiological and social factors play a role in the association between BMI and all-cause mortality [39,47]. Cohen et al. [39] found that the Non-Hispanic Black population has lower mortality rates at obesity levels though higher mortality rates at underweight levels than the Non-Hispanic White population; similarly, women are more likely to experience all-cause mortality than men within the same BMI at obesity or underweight levels. WC as a predictor of central obesity is also strongly related to all-cause mortality [48,52,55,56] regardless of BMI levels. There is a dose-response relationship between WC and all-cause mortality; the higher the WC, the higher the risk of all-cause mortality for the same BMI level [51–53,55].

A popular hypothesis to explain the association between cigarette smoking and weight status and the association with all-cause mortality was done by Garrison et al. [57] using the Framingham Study Dataset. Garrison et al. popularized the hypothesis that cigarette smoking was a moderator in the association between weight status and all-cause mortality though this was not replicated by Sempos et al. [58]. Using the same data from Framingham Study, Sempos et al. [58] found that smoking cigarettes is associated with both all-cause mortality and BMI, though not a moderator in the association between BMI and all-cause mortality, a finding that has been replicated and found in other studies in the US adult population [43,59]. Therefore, cigarette smoking and weight status are associated between them and with all-cause mortality though not necessarily through an interaction.

Nonetheless, to our knowledge, weight status as a mediator between cigarette smoking and all-cause mortality, especially CPD and all-cause mortality, has not been evaluated. A mechanism that supports weight status as a potential mediator at the population level in the association between CPD and all-cause mortality is that the higher the CPD, the higher the levels of central obesity, when approximated by WC [10,16,17,20,22,27], regardless of the direction of the association between the CPD and BMI; central obesity as well as CPD are strongly associated with all-cause mortality [48,55]. In this study, we hypothesized that weight status explains at the population level, at least partially, the association between CPD and all-cause mortality in the population of US adults who currently smoke cigarettes.

The objective of this study was to determine whether weight status, assessed by BMI or WC, mediates the association between CPD and all-cause mortality. Using NHANES, a nationally representative, cross-sectional survey data from the US adult population, we specifically aimed to 1) characterize cigarette smoking, weight status, and all-cause mortality and 2) quantify the association between CPD, weight status, and all-cause mortality.

## Methods

### Study population

We harnessed the 2003-2018 NHANES, which includes US adults (18+). Every NHANES Survey Cycle consists of two years (e.g., 2003-2004), and participants were included by year. NHANES includes data such as biomarkers, demographics, health behaviors (i.e., CPD, alcohol consumption, and physical activity), and measurements (i.e., BMI, WC, and body fat percentage). The mortality data of NHANES’s participants came from the National Center of Health Statistics (NCHS) data linkage, Linked Mortality Files (LMF). The LMF included a limited set of mortality data for adult participants, including follow-up from 2003 to 2019 or until death occurred, whichever was first [60]; multiple survey cycles of NHANES merged with LMF were used to ensure temporality between past 30-day cigarette smoking and all-cause mortality.

**Fig 1:**
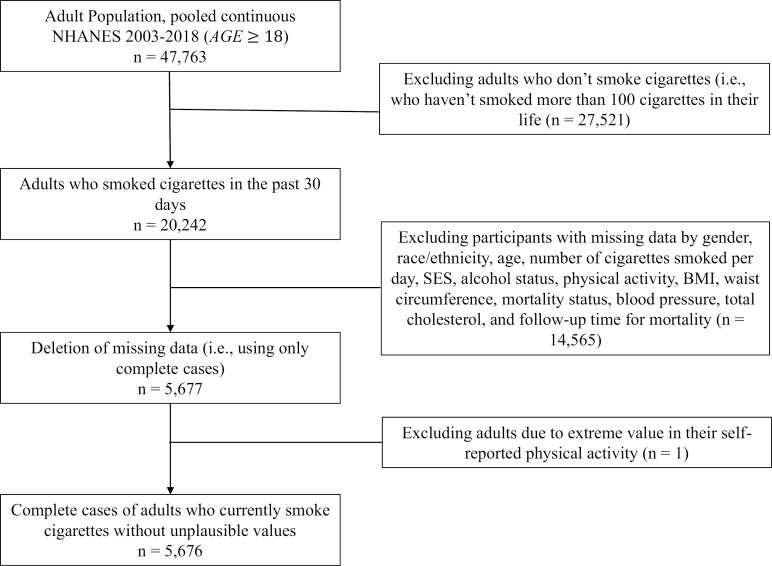
Flow chart of the sample size of adults with past 30-day cigarette smoking in the US from the continuous NHANES, 2003-2018.

We included US adults (+18 years) who had smoked more than one hundred cigarettes and who smoked cigarettes in the past 30 days. We did not include those who formerly smoked cigarettes due to not having data about the CPD in the past 30-days. Fig 1 summarizes the combined NHANES from 2003 to 2018 and LMF of mortality follow-up from 2003 to 2019 of the adult population from the US. We excluded only one participant due to having extreme values in their self-reported physical activity (the participant reported a value of 908 min/day)*.* The other participants were excluded due to missing data from all considered factors (n = 14,565 of adults with past 30-day smoking), resulting in a final analytic sample of n =  5,676, representing 29,862,731 US adults with past 30-day smoking. This study was approved by the IRB of Indiana University (IRB Protocol #16545).

### Scenario

The objectives were accomplished through a mediation analysis in the following scenario: those who currently smoke cigarettes started to smoke cigarettes before the weight status reported at the moment of the interview (verifiable with the comparison of age when starting to smoke cigarettes regularly with the age at the moment of the interview). Therefore, current cigarette smoking occurred first than the weight status at the moment of the interview, and it was assumed, together with weight status, to be fixed during the follow-up time of all-cause mortality.

### Measurements

#### Cigarette smoking (exposure).

CPD was collected through “*During the past 30 days, on the days that you smoked, about how many cigarettes did you smoke per day?”*

#### Weight status (mediator).

Weight status, measured by BMI, was calculated as weight (kg)/height^2^ (m^2^) by the NHANES staff using in-person weight and height measurements. NHANES staff measured WC (cm) using a steel tape while the participant was standing and holding the gown up; the waist level was defined at the abdomen [61]. We used BMI and WC as surrogate measures of weight status for these analyses because lean mass and visceral tissue are important considerations for health outcomes such as all-cause mortality. Lean mass and visceral tissue are included in BMI and WC but are not measured by body fat percentage [62]. Yet, we also included body fat percentages (Android and Gyroid) as surrogate measures of weight status in our analyses to compare results. Body fat percentages (both Android and Gynoid) were measured using a dual-energy ray absorptiometry (DXA) for those without pregnancy and the bioelectrical impedance analysis (BIA) for those who were pregnant at the moment of the interview [61].

#### All-cause mortality (outcome).

Mortality status was a time-to-event variable that indicated whether the participant had died in the study period. Together with mortality status, follow-up time (in years) was used. The follow-up time was measured as the time from the moment of the follow-up interview at the mobile examination center until 2019 or until the participant was reported dead, whichever occurred first.

#### Background characteristics (covariates).

Factors that might influence the association between cigarette smoking and all-cause mortality, especially CPD and all-cause mortality, were included as covariates. Cigarette smoking, weight status, and all-cause mortality vary by sex/gender, age, race/ethnicity, and SES [36,39,43,47,54], as well as physical activity and alcohol consumption [25,28,29,63]. Biomarkers such as blood pressure status and total cholesterol are directly related to all-cause mortality [45,64].

Sex/gender was a dichotomous variable indicating whether the participant self-identified as male or female. Age was a continuous variable that ranged from 18 to 85 years old. The age of participants older than 85 years old was recorded as 85 years old to preserve confidentiality. Race/ethnicity was a polychotomous categorical variable that included the following: Hispanic Mexican, Hispanic Other (i.e., besides Mexican heritage), Non-Hispanic White, Non-Hispanic Black, and Non-Hispanic Other. SES was defined as the *ratio of family income to poverty*, a continuous variable that ranged from zero to five, where values greater than five were given a value of five by NHANES staff; the closer the value was from zero, the higher the poverty level of the participant [65]. Physical activity was operationalized as the total moderate physical activity per day through the sum of minutes per day of walking or using the bicycle, the minutes per day of moderate physical activity, and the minutes per day of vigorous physical activity times two [66]. Alcohol consumption was defined as the average number of drinks drank per week. Blood pressure and total cholesterol were dichotomous variables that indicated whether the participant had a systolic blood pressure of  ≥  120 mm Hg and a diastolic blood pressure <  80 mm Hg or whether the total cholesterol was  ≥  200 mg/dL, respectively.

### Statistical analysis

We did all analyses in R Studio; we used the *survey* package to adjust data based on their sampling design and weights and to construct the models for the mediation analysis. We used the *mediation* package for the mediation analysis. Descriptive statistics of the merged NHANES dataset were adjusted by the survey weights of the mobile examination center divided by the number of survey cycles as recommended by NHANES guidelines [67].

The mediation analysis used bootstrapping with 1,000 simulations to obtain uncertainty estimates around the parameter estimates. We ran 1,000 simulations because mediation estimates converged (i.e., there was no meaningful variability among the estimates of interest). Bootstrapping allows to measure the confidence intervals for such statistical estimates without making any assumption about its distribution (i.e., non-parametric), and it is known to be a robust method with a low risk of biased results*.* An outcome-exposure model and a mediator-exposure model were constructed for the mediation analysis to calculate the indirect, direct, and total effects. We used an AFT model with a Weibull distribution for the outcome-exposure model. We used the AFT Model with a Weibull Distribution because the Cox PH Model assumption, proportionality of hazards, was unmet for some of the covariates, which can produce biased results*.*

Based on the model selection criterion (AIC and BIC), the AFT Model with a Weibull Distribution was the best fit for these data compared to the Cox PH Model for all surrogate measures of weight status. Results obtained from the AFT Model with a Weibull Distribution can be interpreted as a Cox PH Model. The AFT model included the covariates related to all-cause mortality. Due to the evidence of a U-shaped relation between BMI and the risk of all-cause mortality [11,39,41,43,44,46], a quadratic term of BMI was included when BMI was used as the surrogate measure of weight status. For WC, we did not include a quadratic term due to the dose-response relationship between WC and all-cause mortality being somewhat linear [48,55,56].

We constructed the mediator-exposure model using a generalized linear model with a Gaussian distribution, including covariates associated with both CPD and weight status. Based on Cohen et al. [39], BMI was moderated by race/ethnicity and sex/gender and thus, interactions of these covariates were included; for WC, such interactions were not included. The surrogate measure of weight status, body fat percentages (Android and Gynoid), were also considered for the mediation analyses using the same rationale for WC due to also being related to fat mass.

As in previous studies [20,23,48], we did an analysis to adjust WC by BMI levels. Regardless of the change in BMI by CPD, the WC, and thus central obesity, increases as the CPD increases. Such mediation analysis included a quadratic BMI term in the all-cause mortality-cigarette smoking, though only a BMI term in the waist-circumference-cigarette smoking model. Although BMI and WC are highly correlated, WC for a given BMI is a strong predictor of all-cause mortality [68,69]. Furthermore, WC is a measurement of central obesity, while BMI is a measurement of general obesity. Therefore, a person with a certain weight status (not necessarily obesity) can have central obesity. Thus, by adjusting WC for different BMI levels, we can assess central obesity in different weight statuses by BMI.

For sensitivity analyses, we verified the assumption for mediation analysis, sequential ignorability. First, based on prior knowledge, we identified potential confounders, such as alcohol consumption, sex/gender, age, blood pressure, total cholesterol, race/ethnicity, physical activity, and SES. Using different non-parametric association/correlation tests depending on whether the variables were continuous, categorical, or a mix, it was verified whether such potential confounders were actual confounders.

## Results

### Descriptive statistics

Table 1 describes some characteristics of the final sample of adults who smoked cigarettes for the past 30 days from NHANES from 2003 to 2018 (n = 5,676). For example, the weighted mean of CPD was 13.21. As expected, the weighted mean of BMI was in the overweight category, and most participants engaged in non-heavy smoking ( < 20 CPD). The mean survival time was 8.00 years among those who died during the study timeframe (i.e., all-cause mortality event occurred). All-cause mortality occurred among less than 10% of the sample. The association between WC and BMI indicates that both surrogate measures are strongly correlated (see S1 File).

**Table 1:** Descriptive characteristics of sample from NHANES 2003-2018 (n = 5,676).

Sample Characteristics	Weighted mean (SE)
Cigarettes smoked per day (CPD)	13.21 (0.245)
BMI (kg/m^2^)	27.97 (0.125)
Age in years (when interviewed)	42.70 (0.259)
Physical Activity (min/day)	54.58 (1.552)
Alcohol Consumption (Drinks/week)	7.86 (0.292)
SES (USD/poverty measure)	2.35 (0.041)
Mean Survival Time (years)	8.00 (0.139)
Waist Circumference (cm)	97.58 (0.314)
Android Body Fat (%)	34.21 (0.250)
Gynoid Body Fat (%)	34.79 (0.228)
**All-cause Mortality**	**n (weighted %)**
Alive	5,065 (91.47)
Dead	611 (8.53)
**Sex/gender**	**n (weighted %)**
Men	3,253 (54.35)
Women	2,423 (45.65)
**Race/Ethnicity**	**n (weighted %)**
Hispanic (Mexican)	610 (6.10)
Hispanic (Other)	392 (4.22)
Non-Hispanic White	2,855 (70.32)
Non-Hispanic Black	1,353 (12.24)
Non-Hispanic Other	466 (7.12)
**Blood Pressure**	**n (weighted %)**
Systolic ≥ 120 mm Hg and Diastolic < 80 mm Hg	2,661 (49.81)
Systolic < 120 mm Hg and Diastolic ≥ 80 mm Hg	3,015 (50.19)
**Total Cholesterol**	**n (weighted %)**
≥ 200 mg/dL	2,323 (41.94)
< 200 mg/dL	3,353 (58.06)
**BMI Categories**	**n (weighted %)**
Underweight (BMI < 18.5)	170 (2.81)
Normal Weight (18.5 ≤ BMI < 25)	1,908 (34.30)
Overweight (25 ≤ BMI < 30)	1,739 (30.59)
Obesity (BMI ≥ 30)	1,859 (32.29)

### Mediation analysis

**Fig 2:**
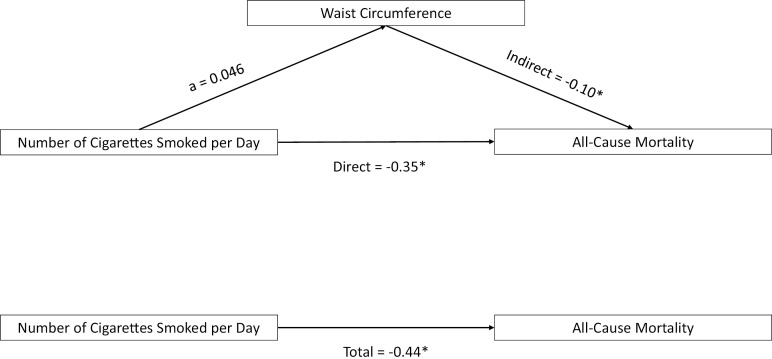
Visualization of mediation analysis with waist circumference. NOTE: values with “*” indicate that estimates are non-linear due to coming from the AFT Model. The “a” value was the estimate effect of CPD of the Waist Circumference- Number of Cigarettes smoked per day model.

The direct effect and total effect were statistically significant for all surrogate measures (BMI, WC, and body fat percentages), indicating that CPD was directly associated with all-cause mortality, as expected (p-values < 0.05; see Table 2). The indirect effects for all surrogate measures were not significant (see Table 2), indicating that weight status did not explain the association between cigarette smoking and all-cause mortality. However, in the mediation analysis where WC was the mediator adjusted by BMI levels, the WC’s indirect effect was statistically significant (p-value <  0.01; see Table 3 and Fig 2) and 21% of the total effect. Thus, WC may partially explain the association between the CPD and all-cause mortality per BMI levels (see Table 3 and Fig 2).

**Table 2:** Results of mediation analysis by surrogate measure of weight status between cigarette smoking and all-cause mortality, NHANES 2003-2018.

Effect/Surrogate Measure	BMI^a^	Waist Circumference	Body Fat % (Android)^b^	Body Fat % (Gynoid)^b^
**Total**	-0.44	-0.46	-1.39	-1.46
	95% CI: (-2.24, -0.22)	95% CI: (-2.15, -0.21)	95% CI: (-4.50, -0.64)	95% CI: (-5.63, -0.83)
	p = 0.0200^*^	p = 0.0180^*^	p = 0.004^*^	p < 0.001^*^
**Indirect**	-0.014	-0.07	0.004	0.10
	95% CI: (-0.006, 0.050)	95% CI: (-0.12, 0.00)	95% CI: (-0.01, 0.11)	95% CI: (-0.04, 0.23)
	p = 0.6900	p = 0.0680^c^	p = 0.7880	p = 0.4300
**Direct**	-0.43	-0.38	-1.39	-1.57
	95% CI: (-2.25, -0.22)	95% CI: (-2.11, -0.17)	95% CI: (-4.58, -0.67)	95% CI: (-5.76, -0.91)
	p = 0.0200^*^	p = 0.0200^*^	p = 0.004^*^	p < 0.001^*^

* p-value <  0.05

^a^All-Cause Mortality-Cigarette Smoking Model included a quadratic term for BMI and the interaction of sex/gender- race/ethnicity

^b^Sample size for Mediation analysis was **2,368** due to higher missing values for Body Fat Percentage (Android and Gynoid) and only considering NHANES 2003-2004 and NHANES 2005-2006

^c^p-value <  0.10

**Table 3:** Results of mediation analysis of WC between cigarette smoking and all-cause mortality adjusted by BMI, NHANES 2003-2018.

Effect	Estimate	95% CI	p-value
**Total**	-0.45	(-2.00, -0.20)	0.0160^*^
**Indirect**	-0.10	(-0.23, -0.05)	<0.001^*^
**Direct**	-0.35	(-1.86, -0.10)	0.0360^*^
**Proportion Mediated**	0.21	(0.07, 1.25)	0.0160^*^

* p-value <  0.05

NOTE: The model for the mediation analysis included BMI as a covariate for the all-cause mortality-cigarette smoking model and the waist circumference-cigarette smoking model. For the all-cause mortality-cigarette smoking model, a quadratic term for BMI was included.

Table 3 shows how changes in WC and CPD influence survival time related to all-cause mortality among US adults who currently smoked cigarettes at the moment of interview but died throughout the follow-up. Specifically, the total effect means that for each unit increase in both CPD and WC, the survival time decreases by 0.45 years, by BMI levels and after adjusting for other covariates. The direct effect focuses only on CPD, indicating that for each unit increase in CPD, survival time decreases by 0.35 years when adjusted for other covariates. The indirect effect examines only WC and shows that for each unit increase in WC, survival time decreases by 0.10 years of time, assuming CPD doesn’t change. In summary, increasing both CPD and WC has a notable negative impact on survival time, meaning that participants were more likely to experience all-cause mortality. Fig 2 visualizes mediation effects among CPD, WC, and all-cause mortality by BMI levels. The coefficient “a” is the effect estimate of WC on CPD from the exposure-mediator model. The “a” indicates that for every additional CPD among those who currently smoke cigarettes, on average, their WC will increase by 0.046 cm. However, the mediation effects and the “a” coefficient are not linear.

## Discussion

This study found that WC was a partial mediator in the association between CPD and all-cause mortality when adjusted by BMI levels at the population level. To our knowledge, this is the first study to assess whether weight status mediates the association between CPD and all-cause mortality. However, WC did not mediate the association between CPD and all-cause mortality when not adjusted by BMI. These findings have two interpretations: (a) obesity, especially central obesity (when approximated by WC), partially mediates the association between CPD and all-cause mortality when adjusted by BMI levels. (b) High levels of correlation between BMI and WC at the population level might bias the results found in this study. We encourage more research to replicate these findings.

To our knowledge, this is the first study to address whether weight status mediates the association between CPD and all-cause mortality. Still, many findings from this study echo those in the literature. Cigarette smoking has been positively and strongly associated with all-cause mortality; the effect of cigarette smoking on all-cause mortality reduced the survival time of adults who currently smoke cigarettes [31–35,70–73], where the greater the CPD, the higher the risk of all-cause mortality [32,73].

Nonetheless, this study has some strengths and limitations. This study’s greatest strength is its external validity among US adults who smoked in the past 30-days. To our knowledge, it is the first to address whether weight status mediates the association between CPD and all-cause mortality. The statistical analyses of this study were innovative and more robust than the usual mediation analyses with time-to-event data due to the application of recommended models depending on the scenario to deal with time-to-event data by Burgos-Ochoa et al. [74]. This study applied the deletion method with a high missing data rate, which may lead to biased results if the missing data do not occur entirely at random [75]. Another limitation of this study is that it only considers all-cause mortality as an outcome of death instead of specific-cause mortality such as cancer or cardiovascular diseases. Data from NHANES about cigarette smoking, demographics, physical activity, alcohol consumption, and medical history were all self-reported, which could lead to recall bias.

NHANES dataset comes from a cross-sectional study. A causal relationship cannot be inferred due to a lack of temporality. This limits the interpretations of this study only to the association between CPD and all-cause mortality. However, we can use these data, with carefulness, for mediation analysis if we have a scenario where the directions of these associations are clear (i.e., temporality can occur) [76]. In this study, we assumed participants started to smoke cigarettes before their reported surrogate measures of weight status, which we verified by comparing the age of starting to smoke cigarettes with age at interview. This scenario allowed us to see the temporality between cigarette smoking and weight status, assuming such surrogate measures were fixed until the moment of the interview. However, we did not consider changes through time in smoking behaviors or weight changes, which might contribute to the risk of all-cause mortality [77].

Doing a mediation analysis with a time-to-event outcome can apply to scenarios where exposure, mediation, or covariates can be either time-dependent or non-time dependent as done by Lange and Hansen [78], a scenario not evaluated by the work of Maxwell, Cole, and Mitchell [79]. As long as type I error is not inflated, there is a lower risk of bias when estimating a mediation effect when using cross-sectional data, though carefulness in the interpretation of results is encouraged [80]. However, given that the AFT model is known to be robust against measurement error, the type I error is likely not to be inflated, and results are less likely to be biased [81].

The findings from this study support promoting weight management programs as a harm reduction approach to reduce the risk of all-cause mortality due to past 30-day cigarette smoking. Weight management programs should be focused on lowering WC levels and, thus, central obesity through promoting more physical activity and less alcohol consumption, especially among those engaging in heavy smoking [27,82]. In case of severe obesity, bariatric surgery should also be used as a weight management intervention to lower WC levels and, therefore, to reduce all-cause mortality due to the CPD [83].

In conclusion, this study provides evidence that supports WC, when adjusted by BMI levels, is a partial mediator in the association between CPD and all-cause mortality. However, more research is needed. Future work should consider other potential sources of bias and confounders based on prior knowledge, such as changes through time in cigarette smoking and weight using longitudinal data and other substances. We also recommend future longitudinal studies to examine and evaluate potential causes of death related to smoking and weight status using other surrogate measures such as visceral fat.

### Implications

Waist circumference, as a surrogate measure of weight status, was a partial mediator between cigarette smoking and all-cause mortality when adjusted by BMI levels. Reducing waist circumference and central obesity through weight loss programs may reduce mortality due to the number of cigarettes smoked per day among adults who smoke.

## Supporting information

S1 File Correlation between body mass index and waist circumference, NHANES 2003-2018. (TIFF)

## Abbreviations

AFT: Accelerated Failure Time
BMI: Body Mass Index
CPD: cigarettes per day
LMF: Linkage Mortality Files
NCHS: National Center Health Statistics
NHANES: National Health and Nutrition Examination Survey
SES: Socioeconomic Status
US: United States
WC: Waist Circumference.
