# Supplementary figures and images for: Determining whether weight status mediates the association between number of cigarettes smoked per day and all-cause mortality among US adults who smoke cigarettes
Source: PLoS One. 2025 Apr 30;20(4):e0319560. doi: 10.1371/journal.pone.0319560 (PMC12043237; doi:10.1371/journal.pone.0319560)

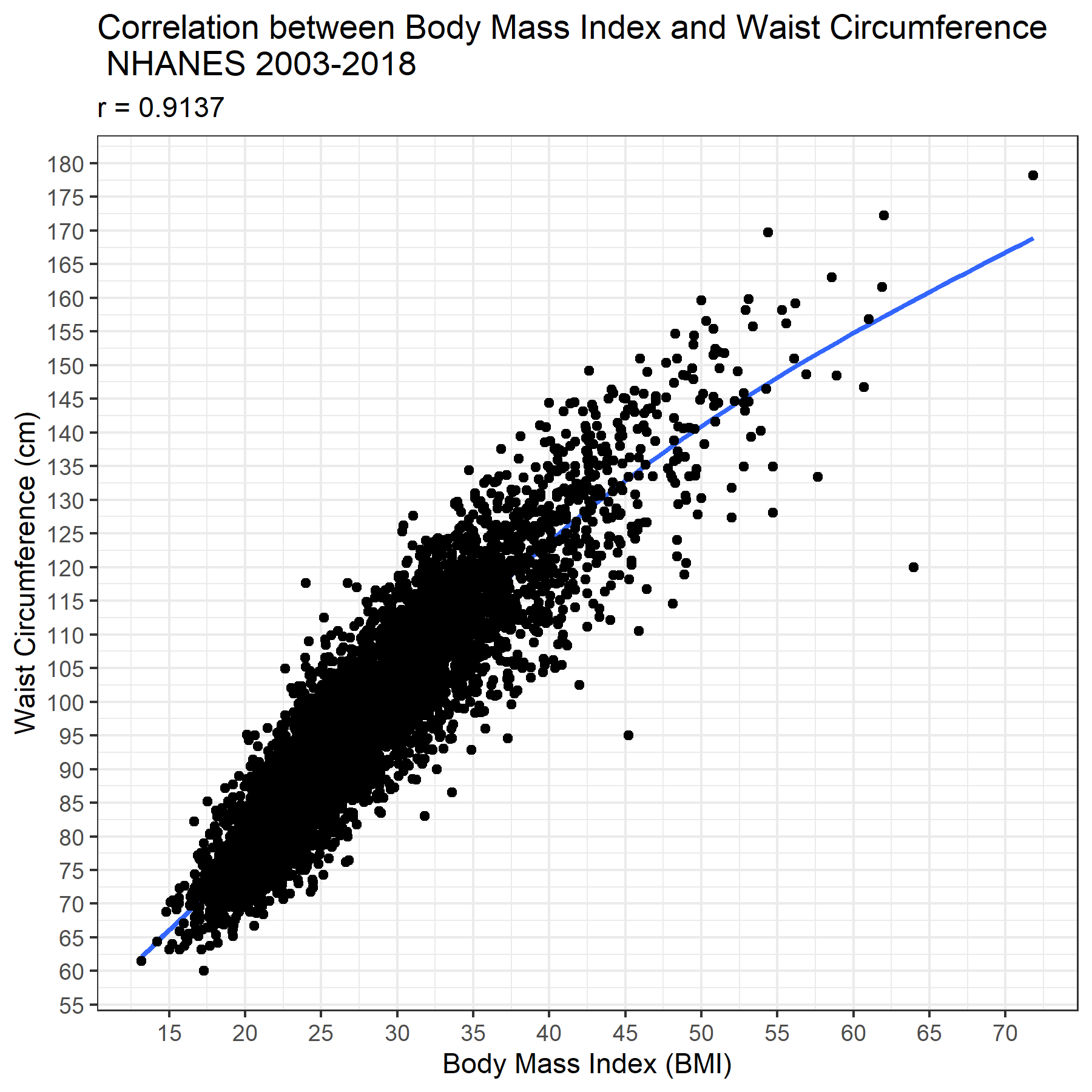

Supplement: S1 File — (TIFF) [file pone.0319560.s001.tiff]
